# Regional and racial outcomes in mortality after atrial fibrillation ablation in medicare Fee for service patients

**DOI:** 10.3389/fcvm.2026.1659282

**Published:** 2026-03-09

**Authors:** Venkata G. Andukuri, Danielle B. Dilsaver, Ryan W. Walters, Liou Xu, Gary Puckrein, Michael H. Kim

**Affiliations:** 1 Department of Medicine, Creighton University and CHI Health, Omaha, NE, United States; 2 National Minority Quality Forum, Washington, DC, United States

**Keywords:** ablation, atrial fibrillation, medicare, mortality, outcomes

## Abstract

**Background:**

Catheter ablation of Atrial Fibrillation (AF) is a cornerstone of treatment. Data on regional and racial variations in AF ablation procedural mortality are limited.

**Methods:**

Data were abstracted from the 2016–2019 Medicare Fee for Service database (FFS), including inpatient and outpatient visits to evaluate regional and racial differences in 30-day AF ablation mortality in Medicare FFS patients. Patients with an AF diagnosis who had AF ablation were identified via ICD-10, CPT, and MS-DRG codes. The primary outcome was between-region (Northeast, Midwest, South, West) differences in 30-day mortality which was assessed using logistic regression models; multivariable models controlled for race, comorbid COPD, and CHA2DS2-VASc score.

**Results:**

38,477 inpatient and 128,544 outpatient AF ablations met inclusion criteria. AF ablation was most common in the South (inpatient: 17,415, outpatient: 55,932) and least in the Northeast which had a 28% lower adjusted odds of 30-day mortality following inpatient AF ablation compared to the South (NE: 1.75.% vs. S: 2.55%; aOR: 0.72, 95% CI: 0.60–0.88) and 23% lower adjusted odds compared to the West (NE: 1.75% vs. W: 2.30%; aOR: 0.81, 95% CI: 0.66–0.99). In outpatients, the Northeast had 44% lower adjusted odds of 30-day mortality compared to the West (NE: 0.15% vs. W:0.25%; aOR 0.56, 95% CI: 0.36–0.87) and the West had a 73% greater odds of 30-day mortality compared to the South (W: 0.25% vs. S: 0.16%; aOR: 1.73, 95% CI: 1.27–2.36). Non-white patients had higher mortality in the outpatient cohort.

**Conclusions:**

Within this Medicare FFS population, significant variations in mortality exist following AF ablation when analyzed across different regions and race. Further research on potential systemic and patient level factors would be of value to help elucidate why these differences are present.

## Introduction

Atrial fibrillation (AF) is the most common cardiac arrhythmia and is associated with an increased risk of stroke, heart failure and mortality (1, 2). Catheter ablation has become a significant evidence-based therapeutic option for the management of AF, particularly in patients with paroxysmal AF and is well supported by clinical trials, registry data, and guidelines (3–6). Despite substantial benefits, catheter ablation carries inherent risks, including procedural mortality. Variation in mortality rates associated with AF ablation have been reported related to patient characteristics, procedural techniques, and operator experience (7). Data are limited regarding regional differences in mortality outcomes (8, 9), inpatient or outpatient mortality variation (9, 10), and racial differences in AF ablation outcomes (9, 11). Higher AF ablation 30-day mortality has been noted in inpatients from 2010 to 2019 (7, 9, 10). Since 2016, the majority of AF ablation procedures are performed in an outpatient classification. This study evaluated both inpatient and outpatient regional and racial differences in 30-day post-catheter ablation mortality among Medicare FFS patients in the United States.

## Methods

### Data source

Study data were abstracted from the 2016 through 2019 Medicare Fee for Service database (FFS), specifically the Medicare institutional claims files that include inpatient, outpatient, hospice, skilled nursing, and home health agency claims. The Medicare FFS database includes demographic, enrollment and payment information, diagnosis and procedure records, and survival data (12). The Medicare FFS is de-identified and HIPAA-compliant. The Institutional Review Board at Creighton University (Info record number: 2004394) acknowledged this study as Not Human Subjects Research.

### Patient cohort

The Medicare patient cohort was identified using inpatient and outpatient files from the Medicare FFS institutional claims data. We included patients at least 18 years old carrying an AF diagnosis who underwent a catheter ablation for AF. First, we identified patients carrying a primary or secondary diagnosis of AF using International Classification of Diseases–Tenth Revision–Clinical Modification codes (ICD-10-CM: I48.0, I48.1, I48.2, or I48.91). For the inpatient AF cohort, we identified patients who had a primary discharge diagnosis for a percutaneous intracardiac procedure (MS-DRG: 273, 274) and who underwent an ablation procedure as indicated by a primary or secondary ICD-10 procedure code (ICD-10-PCS: 02583ZZ). For the outpatient AF cohort, we identified patients carrying a Current Procedural Terminology (CPT) code for atrial fibrillation (AF) ablation (CPT: 93656).

To ensure all ablations were specifically for AF, we excluded patients carrying a diagnosis of supraventricular tachycardia, ventricular tachycardia, atrial flutter, other premature beats, Wolf-Parkinson-White, Lown-Ganong-Levine, atrioventricular nodal tachycardia, and a history of pacemaker. Additionally, we excluded patients who underwent pacemaker implantation, implantable cardioverter-defibrillation implantation, and/or open surgical ablation on the same day as their ablation procedure (see Supplementary Table S1 for a complete list of inclusion/exclusion criteria).

### Patient and visit characteristics

For each visit, we extracted patient-specific demographics that included age, biologic sex, and race (white, non-white, unknown). We calculated patient-specific CHA_2_DS_2_-VASc score that required age, biologic sex, acute myocardial infarction (AMI), congestive heart failure (CHF), hypertension (HTN), stroke, ischemic heart disease, peripheral vascular disease (PVD), and diabetes (13). We also extracted comorbid chronic obstructive pulmonary disease (COPD) and visit-specific characteristics including geographic region (Northeast, Midwest, South, West) (14) geographic division (New England, Middle Atlantic, East North Central, West North Central, South Atlantic, East South Central, West South Central, Mountain, Pacific) (14) and setting (inpatient, outpatient).

### Aims

The primary aim was to evaluate regional differences in 30-day mortality following AF ablation; 30-day mortality was defined as death from any cause within 30 days after AF ablation. Mortality was confirmed from the Medicare denominator files (Medicare Master Beneficiary Summary File). To allow for consistent, complete 30-day post-ablation follow-up from year to year, the index period was the first 11 months of each calendar year (January 1–November 30). The secondary aim was to evaluate between-race differences in 30-day mortality.

### Statistical analysis

All analyses were assessed separately in the inpatient and outpatient settings as these coded AF ablations represent uniquely different populations.

First, we quantified the frequency of inpatient and outpatient AF ablation procedures among Medicare patients. Next, we stratified demographic and clinical characteristics by region. Categorical variables were presented as frequency and percent, and continuous variables were presented as mean and standard deviation. Between-region differences in categorical variables were compared using the chi-square test or Fisher's exact tests, depending on cell counts. Between-region differences in continuous variables were compared using one-way ANOVA. Unadjusted and adjusted logistic regression models were estimated to evaluate region and race differences in the odds of 30-day mortality. Adjusted logistic regression models controlled for race, comorbid COPD, and CHA_2_DS_2_-VASc score.

Analyses were conducted using SAS v. 9.4 with two-tailed *p* < .05 indicating statistical significance.

## Results

### Cohort characteristics

From 2016 to 2019, 38,477 inpatient and 128,544 outpatient AF ablation procedures met the inclusion criteria. Table 1 presents the frequency of AF ablation by sex, race, geographic region, and geographic division in the inpatient and outpatient setting. The greatest number of AF ablation were performed in the South (inpatient: 17,415, outpatient: 55,932). Within the South, the South Atlantic geographic division performed the most AF ablation at 10,447 inpatients and 30,357 outpatients. AF ablation patients were more frequently male and white (Table 1). Table 2 presents age, sex, race, and comorbidities by geographic region. In the inpatient and outpatient setting, hypertension was the most frequent comorbidity, followed by ischemic heart disease and CHF (Table 2). The average CHA_2_DS_2_-VASc score was 4.70 (SD: 1.51) and 3.91 (SD: 1.48) in the inpatient and outpatient settings, respectively (Table 2).

**Table 1:** Frequency of atrial fibrillation ablations.

Demographic characteristics	Inpatient	Outpatient
	*N*	*N*
Overall	38,477	128,544
Sex		
Male	21,989	74,661
Female	16,488	53,883
Race		
White	33,780	119,068
Non-white	3,973	6,135
Unknown	724	3,341
Region		
Northeast	7,987	18,186
Midwest	7,863	26,460
South	17,415	55,932
West	5,212	27,966
Division		
New England	1,685	4,665
Middle Atlantic	6,302	13,521
East North Central	5,667	18,369
West North Central	2,196	8,091
South Atlantic	10,447	30,357
East South Central	2,250	9,523
West South Central	4,724	16,052
Mountain	1,997	9,888
Pacific	3,215	18,078

**Table 2:** Inpatient and outpatient atrial fibrillation ablation descriptives stratified by geographic region.

Patient characteristics	*Overall*	Northeast	Midwest	South	West	*P*
Inpatient, *N*	38,477	7,987	7,863	17,415	5,212	-
Age, years	73.20 ± 8.66	72.86 ± 8.53	72.94 ± 8.77	73.28 ± 8.66	73.86 ± 8.61	<0.001
Biological Sex, %						
Male	21,989 (57.15)	4,787 (59.93)	4,434 (56.39)	9,741 (55.93)	3,027 (58.08)	<0.001
Female	16,488 (42.85)	3,200 (40.07)	3,429 (43.69)	7,674 (44.07)	2,185 (41.92)	
Race, %						
White	33,780 (87.79)	6,933 (86.80)	7,094 (90.22)	15,212 (87.35)	4,541 (87.13)	<0.001
Non-white	3,973 (10.33)	831 (10.40)	608 (7.78)	1,976 (11.35)	558 (10.71)	
Unknown	724 (1.88)	223 (2.79)	161 (2.05)	227 (1.30)	113 (2.17)	
Clinical Characteristics						
CHA_2_DS_2_VASc Score	4.70 ± 1.51	4.62 ± 1.56	4.70 ± 1.46	4.76 ± 1.50	4.66 ± 1.55	<0.001
Chronic Obstructive Pulmonary Disease, %	13,229 (34.38)	2,414 (30.22)	2,988 (38.00)	6,200 (35.60)	1,627 (31.22)	<0.001
Comorbid Congestive Heart Failure, %	26,582 (69.09)	5,261 (65.87)	5,688 (72.34)	12,008 (68.95)	3,625 (69.55)	<0.001
Comorbid Hypertension, %	35,989 (93.53)	7,368 (92.25)	7,397 (94.07)	16,476 (94.61)	4,748 (91.10)	<0.001
Comorbid Stroke, %	3,536 (9.19)	753 (9.43)	626 (7.96)	1,650 (9.47)	507 (9.73)	<0.001
Comorbid Ischemic Heart Disease, %	29,227 (75.96)	6,056 (75.92)	5,985 (76.12)	13,428 (77.11)	3,758 (72.10)	<0.001
Comorbid Peripheral Vascular Disease, %	^a^	^a^	^a^	^a^	^a^	^a^
Comorbid Acute Myocardial Infarction, %	2,418 (6.28)	497 (6.22)	538 (6.84)	1,095 (6.29)	288 (5.53)	0.026
Comorbid Diabetes, %	16,286 (42.33)	3,440 (43.07)	3,330 (42.35)	7,485 (42.98)	2,031 (38.97)	<0.001

Outpatient, N	128,544	18,186	26,460	55,932	27,966	-
Age, years	71.93 ± 6.22	71.53 ± 6.26	71.27 ± 6.20	72.08 ± 6.22	72.51 ± 6.16	<0.001
Biological Sex, %						
Male	74,661 (58.08)	10,730 (59.00)	15,378 (58.12)	32,029 (57.26)	16,524 (59.09)	<0.001
Female	53,883 (41.92)	7,456 (41.00)	11,082 (41.88)	23,903 (42.74)	11,442 (40.91)	
Race, %						
White	119,068 (92.63)	16,638 (91.49)	24,824 (93.82)	52,068 (93.09)	25,538 (91.32)	<0.001
Non-white	6,135 (4.77)	862 (4.74)	872 (3.30)	2,750 (4.92)	1,651 (5.90)	
Unknown	3,341 (2.60)	686 (3.77)	764 (2.89)	1,114 (1.99)	777 (2.78)	
Clinical Characteristics						
CHA_2_DS_2_VASc Score	3.91 ± 1.48	3.89 ± 1.51	3.83 ± 1.45	4.03 ± 1.46	3.77 ± 1.51	<0.001
Chronic Obstructive Pulmonary Disease, %	22,141 (17.22)	3,074 (16.90)	4,849 (18.33)	10,295 (18.41)	3,923 (14.03)	<0.001
Comorbid Congestive Heart Failure, %	55,283 (43.01)	7,284 (40.05)	10,926 (41.29)	22,494 (40.22)	10,579 (37.83)	<0.001
Comorbid Hypertension, %	110,338 (85.84)	15,400 (84.68)	22,570 (85.30)	49,750 (88.95)	22,618 (80.88)	<0.001
Comorbid Stroke, %	7,957 (6.19)	1,171 (6.44)	1,329 (5.02)	3,681 (6.58)	1,776 (6.35)	<0.001
Comorbid Ischemic Heart Disease, %	78,352 (60.95)	11,423 (62.81)	15,796 (59.70)	36,089 (64.52)	15,044 (53.79)	<0.001
Comorbid Peripheral Vascular Disease, %	^a^	^a^	^a^	^a^	^a^	^a^
Comorbid Acute Myocardial Infarction, %	3,064 (2.38)	342 (1.88)	473 (1.79)	881 (1.58)	368 (1.32)	<0.001
Comorbid Diabetes, %	37,164 (28.91)	5,473 (30.09)	7,517 (28.41)	17,258 (30.86)	6,916 (24.73)	<0.001

^a^ indicates a result could not be presented due to insufficient counts per the CMS Data Use Agreement.

Categorical variables were presented as count (percent) and compared via chi-square or Fisher's exact test.

Continuous descriptives were presented as mean ± standard deviation and compared via one-way ANOVA.

Patients receiving inpatient AF ablations in the Northeast and West had lower CHA_2_DS_2_VASc scores compared to the Midwest and South (*p* < 0.001; Northeast: 4.62 ± 1.56, West: 4.66 ± 1.55, Midwest: 4.70 ± 1.46, South: 4.76 ± 1.50). Notably, the West had the lowest rate of comorbid HTN, ischemic heart disease, AMI, and diabetes among inpatient AF ablation patients (Table 2). Similarly, in the outpatient setting, patients receiving care in the West had the lowest CHA_2_DS_2_VASc scores (*p* < 0.001; West: 3.77 ± 1.51, Northeast: 3.89 ± 1.51, Midwest: 3.83 ± 1.45, South: 4.03 ± 1.46). The rate of COPD, CHF, HTN, ischemic heart disease, AMI, and diabetes were lowest in patients receiving outpatient AF ablations in the West (Table 2).

### Regional and racial differences in 30-day mortality

Table 3 presents the 30-day mortality associated with AF ablation by sex, race, geographic region, and geographic division in the inpatient and outpatient setting. The outcome of biological sex differences was not a focus of our study, however, an increased inpatient 30-day mortality was noted among males compared to females.

**Table 3:** 30-day mortality following atrial fibrillation ablation by patient descriptives.

Demographic characteristics	Inpatient			Outpatient		
	*N*	*%*	*p*	*N*	*%*	*p*
Overall	879	2.28	-	243	0.19	-
Sex						
Male	567	2.58	<0.001	152	0.20	0.158
Female	312	1.89		91	0.17	
Race						
White	752	2.23	0.095	212	0.18	0.004
Non-white	110	2.77		22	0.36	
Unknown	17	2.35		9.00	0.27	
Region						
Northeast	140	1.75	0.001	28	0.15	0.026
Midwest	175	2.23		52	0.20	
South	444	2.55		92	0.16	
West	120	2.30		71	0.25	
Division						
New England	40	2.37	<0.001	^a^	^a^	0. 136
Middle Atlantic	100	1.59		24	0.18	
East North Central	121	2.14		35	0.19	
West North Central	54	2.46		17	0.21	
South Atlantic	254	2.43		48	0.16	
East South Central	75	3.33		20	0.21	
West South Central	115	2.43		24	0.15	
Mountain	48	2.40		24	0.24	
Pacific	72	2.24		47	0.26	

^a^ indicates the result could not be presented due to insufficient counts per the CMS Data Use Agreement.

Categorical variables were compared via chi-square tests.

### Inpatient

Of the inpatient AF ablation patients, 2.28% (*N* = 879) died within 30 days of their procedure. The Northeast had the lowest mortality rate following inpatient AF ablation at 1.75% (*N* = 140), followed by the Midwest at 2.23% (*N* = 175) and the West at 2.30% (*N* = 120). The South had the highest mortality rate at 2.55% (*N* = 444). Adjusted for race, comorbid COPD, and CHA_2_DS_2_-VASc score, patients undergoing AF ablation in the Northeast had 28% lower adjusted odds of 30-day mortality compared to patients treated in the South (*N*E: 1.75.% vs. S: 2.55%; aOR: 0.72, 95% CI: 0.60–0.88, *p* = 0.001; Table 4) as well as a 23% lower adjusted odds of 30-day mortality compared to patients undergoing AF ablation in the West (NE: 1.75% vs. W: 2.30%; aOR: 0.81, 95% CI: 0.66–0.99, *p* = 0.036; Table 4). The adjusted odds of 30-day mortality following inpatient AF ablation were statistically similar between the Northeast and Midwest, Midwest and South, West and South, and Midwest and West (Table 4).

**Table 4:** Adjusted odds of 30-day mortality following atrial fibrillation ablation by region and race.

Comparison of Inpatient/Outpatient	Unadjusted Rate	Adjusted Odds Ratio (95% CI), *p*-value
Inpatient		
Region		
Northeast v. South	1.75% vs. 2.55%	0.72 (0.60–0.88), *p* = 0.001
Midwest v. South	2.23% vs. 2.55%	0.88 (0.74–1.05), *p* = 0.162
West v. South	2.30% vs. 2.55%	0.94 (0.77–1.15), *p* = 0.559
Northeast v. West	1.75% vs. 2.30%	0.77 (0.60–0.99), *p* = 0.038
Midwest v. West	2.23% vs. 2.30%	0.94 (0.74–1.19), *p* = 0.584
Northeast v. Midwest	1.75% vs. 2.23%	0.82 (0.66–1.03), *p* = 0.088
Race		
Non-white v. White	2.77% vs. 2.23%	1.17 (0.96–1.44), *p* = 0.123

Outpatient		
Region		
Northeast v. South	0.15% vs. 0.16%	0.97 (0.64–1.49), *p* = 0.898
Midwest v. South	0.20% vs. 0.16%	1.30 (0.92–1.82), *p* = 0.136
West v. South	0.25% vs. 0.16%	1.73 (1.27–2.36), *p* = 0.001
Northeast v. West	0.15% vs. 0.25%	0.56 (0.36–0.87), *p* = 0.010
Midwest v. West	0.20% vs. 0.25%	0.75 (0.52–1.08), *p* = 0.118
Northeast v. Midwest	0.15% vs. 0.20%	0.75 (0.47–1.19), *p* = 0.221
Race		
Non-white v. White	0.36% vs. 0.18%	1.73 (1.11–2.69), *p* = 0.016

CI, Confidence Interval. Reference group follows ‘v.'.

The 30-day mortality following inpatient AF ablation was 2.23% (*N* = 752) for white patients and 2.77% (*N* = 110) for non-white patients. After adjusting for region, comorbid COPD, and CHA_2_DS_2_-VASc score, the adjusted odds of 30-day mortality following inpatient AF ablation were statistically similar between non-white and white patients (Non-white: 2.77% vs. White: 2.23%; aOR: 1.17, 95% CI: 0.96–1.44, *p* = 0.123; Table 4).

### Outpatient

Of outpatient AF ablation patients, 0.19% (*N* = 243) died within 30 days of their procedure. The Northeast had the lowest mortality rate following outpatient AF ablations at 0.15% (*N* = 28), followed by the South at 0.16% (*N* = 92). The West and Midwest had highest 30-day mortality following outpatient AF ablation at 0.25% (*N* = 71) and 0.20% (*N* = 52), respectively. After adjusting for race, comorbid COPD, and CHA_2_DS_2_-VASc score, patients treated in the Northeast were associated with 44% lower adjusted odds of 30-day mortality compared to patients undergoing outpatient AF ablation in the West (aOR 0.56, 95% CI: 0.36–0.87, *p* = 0.01; Table 4). Patients who received an outpatient AF ablation in the West had 73% greater adjusted odds of 30-day mortality compared to patients treated in the South (aOR: 1.73, 95% CI: 1.27–2.36, *p* < 0.001; Table 4). The adjusted odds of 30-day mortality following an outpatient AF ablation were statistically similar between the Northeast and South, Midwest and South, Midwest and West, and Northeast and Midwest (Table 4).

The 30-day mortality following outpatient AF ablation was 0.18% (*N* = 212) for white patients and 0.36% (*N* = 22) for non-white patients. After adjusting for region, comorbid COPD, and CHA_2_DS_2_-VASc score, compared to white patients, non-white patients were associated with 73% greater odds of 30-day mortality following outpatient AF ablation (aOR: 1.73, 95% CI: 1.11–2.69, *p* = 0.016; Table 4).

## Discussion

This study provided additional data on regional and racial differences in AF ablation procedural mortality, both in inpatient and outpatient settings, with stratification by geographic region and divisions and patient demographics in an older, Medicare FFS population. The South region performed the largest number of procedures yet was associated with higher mortality rates. Conversely, the Northeast region performed the fewest procedures and was associated with lower mortality rates. Non-white AF ablation patients had worse mortality in the outpatient cohort. The presence of these significant differences across regions and race is the main finding rather than the actual comparative numbers between regions. These differences highlight the need for an evaluation or discussion of potential reasons for such outcome variation. There may be potential systemic differences in care delivery, access to care, patient selection or population specific burden of comorbidities, performance of AF ablation procedures especially in high volume AF ablation centers, disparities in healthcare, socioeconomic factors, or other unknown confounders.

Both inpatient and outpatient procedures demonstrated unique risk profiles, with inpatient settings generally associated with higher complication and mortality rates. The coding of either inpatient or outpatient status may also reflect system practice patterns or patient specific factors of higher risk. These differences emphasize the need for tailored strategies to address specific risks in each procedural setting.

Within the US, there have been limited data describing the regional differences in 30-day mortality after AF ablation (8). Regional disparities in outcomes may reflect differences in healthcare infrastructure, availability of specialized care, and follow-up practices (7, 15–17). Previous studies have linked procedural outcomes to healthcare system characteristics, with higher volume centers and experienced operators showing significantly better outcomes (7, 15–17). Potential disparities in early diagnosis, delay in treatment of AF, institutional resources, and equitable access to care both prior to and after ablation may have led to worse outcomes and increased 30-day mortality.

Differences in patient demographics and comorbid conditions across regions can influence outcomes. This Medicare FFS study population was older than reported in clinical trials and national registry data (3–6). Patients in the South had higher rates of comorbid conditions, such as hypertension (94.61% inpatient, 88.95% outpatient) and diabetes (42.98% inpatient, 30.86% outpatient). The CHA_2_DS_2_-VASc scores which predict stroke risk in AF patients, were higher in the South. Higher CHA_2_DS_2_-VASc scores and comorbidities such as COPD, hypertension and diabetes, particularly prevalent in the South region, may have contributed to the higher mortality rates observed in this region, even with adjustment of CHA_2_DS_2_-VASc. These findings align with those from Cheng et al. (7), who reported that multiple comorbidities significantly increase risks of adverse events following AF ablation. Unmeasured comorbidities or other confounders may also have played a significant role in outcomes.

Using the Nationwide Inpatient Sample database of 20,085 patients from 2016 to 2019, Aggarwal et al. (11), reported higher in-hospital mortality in black patients with heart failure and preserved ejection fraction following AF ablation. In the current study, non-white patients had higher unadjusted 30-day mortality rates for both inpatient (2.77%) and outpatient (0.36%) procedures compared to white patients. After adjustment for CHA_2_DS_2_-VASc, the difference was non-significant in the inpatient setting. In the outpatient coded AF ablation procedures, the increased risk for non-white patients was significant (adjusted OR: 1.73, 95% CI: 1.11–2.69, *p* = 0.016). Racial disparities in cardiovascular care and outcomes are prevalent. In the prospective ARIC study by Magnani et al. (18), the outcome of AF on the rates of stroke, heart failure (HF), CHD, and mortality was worse in black individuals compared to white individuals. A study by Bhatia et al. (19) showed that non-white patients with HF and AF had a disproportionately higher risk of inpatient death compared with white patients with HF, and there was a significant underutilization of cardioversion and catheter ablation in minority racial groups compared with white patients. Furthermore, Alhuneafat et al. (20) reported that black patients undergoing catheter ablation for AF had higher rates of complications and mortality compared to other patients potentially due to differences in comorbidities and access to care.

This study evaluated Medicare FFS data from all 50 states and the District of Columbia and found AF ablation utilization rates varied substantially from 0.1% to 1.18% and 30-day mortality post-ablation ranged from 0 to 1.21%. These findings could not be analyzed statistically on a state-by-state comparison.

The cost of AF is a significant economic burden worldwide. In addition to the direct cost of AF, individuals with AF have incrementally increased healthcare costs to non-AF cardiovascular and non-cardiovascular expenditures (21). AF ablation has increasing data supporting not only clinical benefit but also beneficial effects in cost-efficiency for managing AF through the reduction of healthcare resource utilization (22).

### Limitations

Despite using inpatient and outpatient data and performing adjustments, the study lacked patient-level data (NYHA class, ejection fraction, medications) and only included older, Medicare FFS patients, which is not fully representative of the AF ablation population. In addition, the Medicare FFS beneficiary files did not include the Medicare Advantage population that has grown significantly in recent years and may limit the generalizability of these findings to the broader Medicare population. The incorporation of Medicare Advantage data in future research would be of value. Regional differences in care could involve other confounding variables and system processes of care that could not be fully adjusted nor measured. Data on non-white patients could not be further sub-categorized due to small numbers. Due to data limitations, race and ethnicity may be conflated. Hispanic patients may be captured in either the white or non-white race categories.

## Conclusion

In summary, this study reveals significant regional and racial disparities in 30-day mortality following AF ablation procedures in Medicare FFS patients. AF ablation is a proven treatment, however, if not applied evenly across a population, this may result in disparities in care and differential outcomes. These data represent another report of increased mortality from real world data which is not seen in clinical trials, NCDR Registry, or clinical centers of excellence (3–7). Clinical trial and registry data demonstrate that the procedure is safe and effective where applied. Real-world data, however, in the Medicare FFS population demonstrate outcomes variation that may help to inform further research on disparate outcomes possibly related to systemic and patient-level factors in different regions.

## Data Availability

Medicare Fee-for-Service (FFS) data can be acquired through the Centers for Medicare & Medicaid Services (CMS) via their Research Data Assistance Center (ResDac). More information can be found at https://resdac.org/cms-fee-information-research-identifiable-data.
