# Supplementary material for: Regional and racial outcomes in mortality after atrial fibrillation ablation in medicare Fee for service patients
Source: Front Cardiovasc Med. 2026 Mar 9;13:1659282. doi: 10.3389/fcvm.2026.1659282 (PMC13006320; doi:10.3389/fcvm.2026.1659282)
Supplement: Supplementary file 1 [file Table1.docx]

| **Supplemental Table 1**. Exclusion criteria diagnosis and procedure codes. | | | |
| --- | --- | --- | --- |
| **Exclusion diagnosis and procedures** | **ICD-10-CM** | **ICD-10-PCS** | **CPT** |
| Supraventricular tachycardia | I47.1 |  |  |
|  | I45.6 |  |  |
| Ventricular tachycardia | I47.2 |  |  |
|  | I47.0 |  |  |
|  | I49.01 |  |  |
|  | I49.02 |  |  |
|  | I49.3 |  |  |
| Atrial flutter | I48.3 |  |  |
|  | I48.4 |  |  |
|  | I48.92 |  |  |
| Other premature beats | I49.3 |  |  |
|  | I49.49 |  |  |
| Wolf-Parkinson-White | I45.5 |  |  |
| Lown-Ganong-Levine | I45.6 |  |  |
| Atrioventricular nodal tachycardia | I45.89 |  |  |
| AV nodal ablation | Z95.0 |  | 93650 |
|  | Z95.810 |  |  |
| Pacemaker Implantation |  | 0JH634Z | 33206 |
|  |  | 0JH636Z | 33207 |
|  |  |  | 33208 |
| Implantable cardioverter- defibrillation implantation |  | 0JH608Z | 33249 |
|  |  | 0JH638Z |  |
|  |  | 0JH808Z |  |
|  |  | 0JH838Z |  |
| Open surgical ablation |  | 02550ZZ | 33250 |
|  |  | 02560ZZ | 33251 |
|  |  | 02570ZZ | 33254 |
|  |  | 02580ZZ | 33255 |
|  |  | 02590ZZ | 33256 |
|  |  | 025F0ZZ | 33257 |
|  |  | 025G0ZZ | 33258 |
|  |  | 025H0ZZ | 33259 |
|  |  | 025J0ZZ | 33261 |
|  |  | 025K0ZZ |  |
|  |  | 025L0ZZ |  |
|  |  | 025M0ZZ |  |
|  |  | 02B50ZZ |  |
|  |  | 02B60ZZ |  |
|  |  | 02B70ZZ |  |
|  |  | 02B80ZZ |  |
|  |  | 02B90ZZ |  |
|  |  | 02BF0ZZ |  |
|  |  | 02BG0ZZ |  |
|  |  | 02BH0ZZ |  |
|  |  | 02BJ0ZZ |  |
|  |  | 02BK0ZZ |  |
|  |  | 02BL0ZZ |  |
|  |  | 02BM0ZZ |  |
|  |  | 02T80ZZ |  |
